# What Are the Long-term Results of MUTARS^®^ Modular Endoprostheses for Reconstruction of Tumor Resection of the Distal Femur and Proximal Tibia?

**DOI:** 10.1007/s11999-015-4644-8

**Published:** 2015-12-09

**Authors:** Michaël P. A. Bus, Michiel A. J. van de Sande, Marta Fiocco, Gerard R. Schaap, Jos A. M. Bramer, P. D. Sander Dijkstra

**Affiliations:** 1 Department of Orthopaedic Surgery, Leiden University Medical Center, Albinusdreef 2, 2300 RC Leiden, The Netherlands; 2 Department of Orthopaedic Surgery, Academic Medical Center, Amsterdam, The Netherlands; 3 Department of Medical Statistics and Bioinformatics, Leiden University Medical Center, Leiden, The Netherlands; 4 The Mathematical Institute, Leiden University, Leiden, The Netherlands

## Abstract

**Background:**

Modular endoprostheses are commonly used to reconstruct defects of the distal femur and proximal tibia after bone tumor resection. Because limb salvage surgery for bone sarcomas is relatively new, becoming more frequently used since the 1980s, studies focusing on the long-term results of such prostheses in treatment of primary tumors are scarce.

**Questions/purposes:**

(1) What proportion of patients experience a mechanical complication with the MUTARS^®^ modular endoprosthesis when used for tumor reconstruction around the knee, and what factors may be associated with mechanical failure? (2) What are the nonmechanical complications? (3) What are the implant failure rates at 5, 10, and 15 years? (4) How often is limb salvage achieved using this prosthesis?

**Methods:**

Between 1995 and 2010, endoprostheses were the preferred method of reconstruction after resection of the knee in adolescents and adults in our centers. During that period, we performed 114 MUTARS^®^ knee replacements in 105 patients; no other endoprosthetic systems were used. Four patients (four of 105 [4%]) were lost to followup, leaving 110 reconstructions in 101 patients for review. The reverse Kaplan-Meier method was used to calculate median followup, which was equal to 8.9 years (95% confidence interval [CI], 8.0–9.7). Mean age at surgery was 36 years (range, 13–82 years). Predominant diagnoses were osteosarcoma (n = 56 [55%]), leiomyosarcoma of bone (n = 10 [10%]), and chondrosarcoma (n = 9 [9%]). In the early period of our study, we routinely used uncemented uncoated implants for primary reconstructions. Later, hydroxyapatite (HA)-coated implants were the standard. Eighty-nine reconstructions (89 of 110 [81%]) were distal femoral replacements (78 uncemented [78 of 89 {88%}, 42 of which were HA-coated [42 of 78 {54%}]) and 21 (21 of 110 [19%]) were proximal tibial replacements. In 26 reconstructions (26 of 110 [24%]), the reconstruction was performed for a failed previous reconstruction. We used a competing risk model to estimate the cumulative incidence of implant failure.

**Results:**

Complications of soft tissue or instability occurred in seven reconstructions (seven of 110 [6%]). With the numbers we had, for uncemented distal femoral replacements, we could not detect a difference in loosening between revision (five of 17 [29%]) and primary reconstructions (eight of 61 [13%]) (hazard ratio [HR], 1.72; 95% CI, 0.55–5.38; p = 0.354). Hydroxyapatite-coated uncemented implants had a lower risk of loosening (two of 42 [5%]) than uncoated uncemented implants (11 of 36 [31%]) (HR, 0.23; 95% CI, 0.05–1.06; p = 0.060). Structural complications occurred in 15 reconstructions (15 of 110 [14%]). Infections occurred in 14 reconstructions (14 of 110 [13%]). Ten patients had a local recurrence (10 of 101 [10%]). With failure for mechanical reasons as the endpoint, the cumulative incidences of implant failure at 5, 10, and 15 years were 16.9% (95% CI, 9.6–24.2), 20.7% (95% CI, 12.5–28.8%), and 37.9% (95% CI, 16.1–59.7), respectively. We were able to salvage some of the failures so that at followup, 90 patients (90 of 101 [89%]) had a MUTARS^®^ in situ.

**Conclusions:**

Although no system has yet proved ideal to restore normal function and demonstrate long-term retention of the implant, MUTARS^®^ modular endoprostheses represent a reliable long-term option for knee replacement after tumor resection, which seems to be comparable to other modular implants available to surgeons. Although the number of patients is relatively small, we could demonstrate that with this prosthesis, an uncemented HA-coated implant is useful in achieving durable fixation.

**Level of Evidence:**

Level IV, therapeutic study.

## Introduction

Various techniques have been described for management of reconstruction of malignant tumors about the knee in adults, including implantation of osteoarticular allografts [25, 34], allograft-prosthetic composites [10, 23] and custom-made [26, 27] or modular [13, 28] endoprotheses. Endoprosthetic reconstruction likely is the most commonly used approach, in part as a result of the ease of use compared with other options and the difficulty of obtaining allografts in some centers in addition to the reported risks of nonunion, fracture, and infection [6, 26, 27]. Potential advantages of endoprostheses include their relative availability, immediate stability, the possibility of rapid recovery, and early weightbearing [26]. Compared with custom-made implants, modular endoprostheses provide the ability to adjust the proper length at the time of the reconstruction [7].

Nevertheless, revisions of endoprosthetic reconstructions occur frequently. Infection, occurring in 6% to 20% of patients, is the leading cause of failure in the early years after surgery [2, 15, 22, 26–28, 32]. In the longer term, mechanical complications are the main concern, most notably aseptic loosening, periprosthetic fractures, and wear [13, 17, 19]. Because the survival of patients with bone sarcomas has improved, and most patients with primary bone tumors are young and active and place high demands on their implants, improving implant designs and reconstructive techniques are essential to reduce the risk of mechanical complications [26]. The MUTARS^®^ system (Modular Universal Tumor And Revision System; implantcast, Buxtehude, Germany; FDA approval pending) was introduced in 1992 and has since been widely used in Europe, Australia, and various Asian countries; results of its use in both orthopaedic oncology and revision surgery have been documented [12, 13, 16]. To our knowledge, no studies have evaluated the intermediate- to long-term results of the MUTARS^®^ knee replacement system in primary tumor reconstructions and revision procedures.

We therefore asked: (1) What proportion of patients experience a mechanical complication with the MUTARS^®^ modular endoprosthesis when used for tumor reconstruction around the knee, and what factors may be associated with mechanical failure? (2) What are the nonmechanical complications? (3) What is the cumulative incidence of implant failure at 5, 10, and 15 years? (4) How often is limb salvage achieved using this prosthesis?

## Patients and Methods

We present a retrospective case series of all patients with a primary malignant or aggressive benign bone or soft tissue tumor in whom a MUTARS^®^ distal femoral or proximal tibial replacement was performed for primary reconstruction or for revision of a failed previous reconstruction. Institutional databases were searched to identify patients who had MUTARS reconstruction between 1995 and 2010 with a minimum followup of 5 years. During the early period under study, we performed a limited number of osteoarticular allograft reconstructions, mainly in young patients. In case it was possible to save adjacent joints, we preferred to perform an intercalary resection and reconstructed the defect with an allograft [5, 6]. Generally speaking, endoprosthetic reconstruction was the preferred method of reconstruction when resection of the knee was deemed inevitable in adolescents and adults. No other endoprosthetic systems have been used in our centers. We performed a total of 114 MUTARS^®^ reconstructions about the knee during the period in question in 105 patients. Four patients (four of 105 [4%]) were lost to followup, leaving 110 reconstructions in 101 patients for review; of these, 64 (64 of 101 [63%]) were alive at final review. The reverse Kaplan-Meier method was used to calculate the median followup, which was equal to 8.9 years (95% confidence interval [CI], 8.0–9.7) (Table 1).

**Table 1:** Study data

Variable	Number	Percent of relevant group
Sex		
Male	55	55
Female	46	45
Diagnosis		
Osteosarcoma	56	55
Leiomyosarcoma of bone	10	10
Chondrosarcoma	9	9
Giant cell tumor of bone	8	8
Pleomorphic undifferentiated sarcoma	7	7
Ewing sarcoma	5	5
Low-grade osteosarcoma	2	2
Sarcoma not otherwise specified	2	2
Synovial sarcoma	1	1
Diffuse-type giant cell tumor	1	1
Reconstruction site		
Distal femur	89	81
Proximal tibia	21	19
Neoadjuvant and adjuvant therapies (around implantation of MUTARS^®^)		
Neoadjuvant chemotherapy	61	60
Adjuvant chemotherapy	64	63
Neoadjuvant radiotherapy	2	2
Adjuvant radiotherapy	4	4
Reconstruction details		
Conventional polyethylene locking mechanism	39	35
PEEK-OPTIMA^®^ locking mechanism	71	65
Extensor reconstruction	19	17
MUTARS^®^ attachment tube used	16	15
Complications		
Type I (soft tissue, instability)	7	6
Type II (aseptic loosening)	17	16
Type III (structural)	15	14
Type IV (infection)	14	13
Type V (tumor progression)	10	10
Failure		
Any type of revision, including refixation	40	36
Major revision/removal entire prosthesis	27	25
Status at final followup		
No evidence of disease	64	63
Alive with disease	–	–
Died of disease	34	34
Died of other cause	3	3

All diagnoses were proven histologically before operation. The feasibility of limb-salvaging resection was evaluated on MRI. In the case of suspected joint involvement, an extraarticular resection was performed removing the joint en bloc with the patella cut in the coronal plane. Of 84 implants (84 of 110 [76%]) that were implanted for primary reconstruction after tumor resection, 39 (46%) had an extraarticular resection. Twenty-six implants (26 of 110 [24%]) were implanted as a revision of a failed reconstruction, including nine MUTARS^®^ and 17 other reconstructions (Table 2).

**Table 2:** Procedures performed before implantation of the primary MUTARS^®^, subsequent reconstructions, and reasons for failure

Procedure	Reconstruction	Number	Reason(s) for reconstruction failure
En bloc resection	Allograft-prosthetic composite	6	Allograft collapse (n = 2), allograft fracture (n = 2), nonunion (n = 1), infection (n = 1)
	Kotz prosthesis	4	Prosthetic fracture (n = 2), loosening (n = 1), infection (n = 1)
	Intercalary allograft	3	Nonunion (n = 2), allograft fracture (n = 1)
	Osteoarticular allograft	2	Allograft fracture
	Extracorporeally radiated autograft	1	Resorption
	Inlay allograft	1	Recurrence
Curettage	Cancellous bone grafting	5	Recurrence
	Cement	3	Recurrence
Arthroplasty	TKA	1	–

A lateral or medial parapatellar approach was used; this depended on the location of the tumor and biopsy tract, which was excised in continuity with the tumor. In all cases, we used a rotating hinged MUTARS^®^ distal femoral or proximal tibial replacement. A polyethylene locking mechanism connected the femoral and tibial components. Until March 2003, we used the conventional polyethylene lock. From then onward, the PEEK-OPTIMA^®^ (Invibio Ltd, Thornton-Cleveleys, UK) lock was used. Extension of the implant was possible in 20-mm increments. All stems and extension pieces were equipped with sawteeth at the junctions to allow rotational adjustment in 5° increments. The hexagonally shaped stems were available for uncemented (TiAl6V4) or cemented (CoCrMo) fixation. Femoral stems were curved to match the natural anterior curvature of the femoral diaphysis. We generally preferred uncemented fixation, unless we were unable to obtain adequate press-fitting or in cases in which bone quality was deemed insufficient for uncemented fixation. In the early period under study, we routinely used uncemented uncoated implants because at that time, the MUTARS^®^ system did not come with hydroxyapatite (HA)-coated stems standardly; HA-coated stems were mainly used in cases with a presumed higher risk of loosening such as patients with a failed previous reconstruction. Later, HA-coated implants were the standard for primary reconstruction. The medullary cavity was reamed with a hexagonal rasp to secure optimal contact between the bone and implant. In case of uncemented fixation, the medullary cavity was underreamed by 1 mm. In case of cemented fixation, we overreamed the canal for 2 mm and third-generation cementing techniques were used.

In cases in which an extensor mechanism reconstruction had to be performed, we ran nonabsorbable sutures through the designated holes in the tibial component to fix an attachment tube (implantcast) to the implant; the extensor mechanism was later attached to the tube, again using nonabsorbable sutures. After assemblage of the prosthesis, a trial reduction was performed. A final check was performed to assess knee motion and soft tissue tension and subsequently, the implant was locked.

All patients received prophylactic intravenous cephalosporins before surgery; these were continued for 1 to 5 days. Drains were removed after a maximum of 48 hours. Based on pain, patients were mobilized under supervision of a physical therapist, usually on the first postoperative day. Antithrombotic prophylaxis was given until 6 weeks postoperatively.

Patients were followed during outpatient visits at 2 and 6 weeks after discharge, after 3 and 6 months, and every 6 months thereafter. Radiographic followup consisted of conventional radiographs and additional imaging (CT/MRI) if complications or recurrence were suspected.

Complications and failures were recorded and classified according to Henderson et al. [17, 18]. Aseptic loosening was defined as migration of the prosthesis on imaging (periprosthetic lucency on conventional radiographs or CT scan or halo formation on CT) in the absence of infection. We however chose to report on the clinical rather than radiological loosening, ie, those that required revision, partly because it can be hard to determine which cases are at risk for future failure/loosening, and it is therefore difficult to reliably comment on the occurrence and significance of these signs. Radiographic signs alone were not observed as a reason for implant failure. Rates of aseptic loosening were compared between primary and revision reconstructions (arthroscopy, curettage, and conventional TKA were not considered as previous reconstructions). Periprosthetic and prosthetic fractures were diagnosed on imaging or intraoperatively. Infection was defined as any deep (periprosthetic) infectious process diagnosed through physical examination, imaging, laboratory tests (including C-reactive protein, erythrocyte sedimentation rate, and synovial fluid leukocyte count) and microbiologic cultures.

### Statistical Analysis

All data were complete. To estimate the cumulative incidence of revision for different types of failure, a competing risks model was used with patient mortality as a competing event [20, 30]. Failures were defined as removal of part of or all of the implant, major revision (exchange of the femoral component, tibial component, or the locking mechanism), or cemented refixation as the endpoint. Failure did not include isolated revision of the bushing. The influence of potential risk factors on the cumulative incidence of revision was determined with Cox regression analyses. SPSS 21.0 (IBM Corp, Armonk, NY, USA) was used for statistical analysis (level of significance, p < 0.050). All analyses for the competing risk models have been performed with the mstate library [9] in the R software package [31].

Mean age at surgery was 36 years (range, 13–82 years). Predominant diagnoses were osteosarcoma (n = 56 [55%]), leiomyosarcoma of bone (n = 10 [10%]), chondrosarcoma (n = 9 [9%]), giant cell tumor of bone (n = 8 [8%]), and pleomorphic undifferentiated sarcoma (n = 7 [7%]). Sixty-four patients (64 of 101 [63%]) were treated with chemotherapy (according to appropriate protocols) around the period of MUTARS^®^ implantation and four (four of 101 [4%]) underwent radiotherapy.

**Fig. 1A–B:**
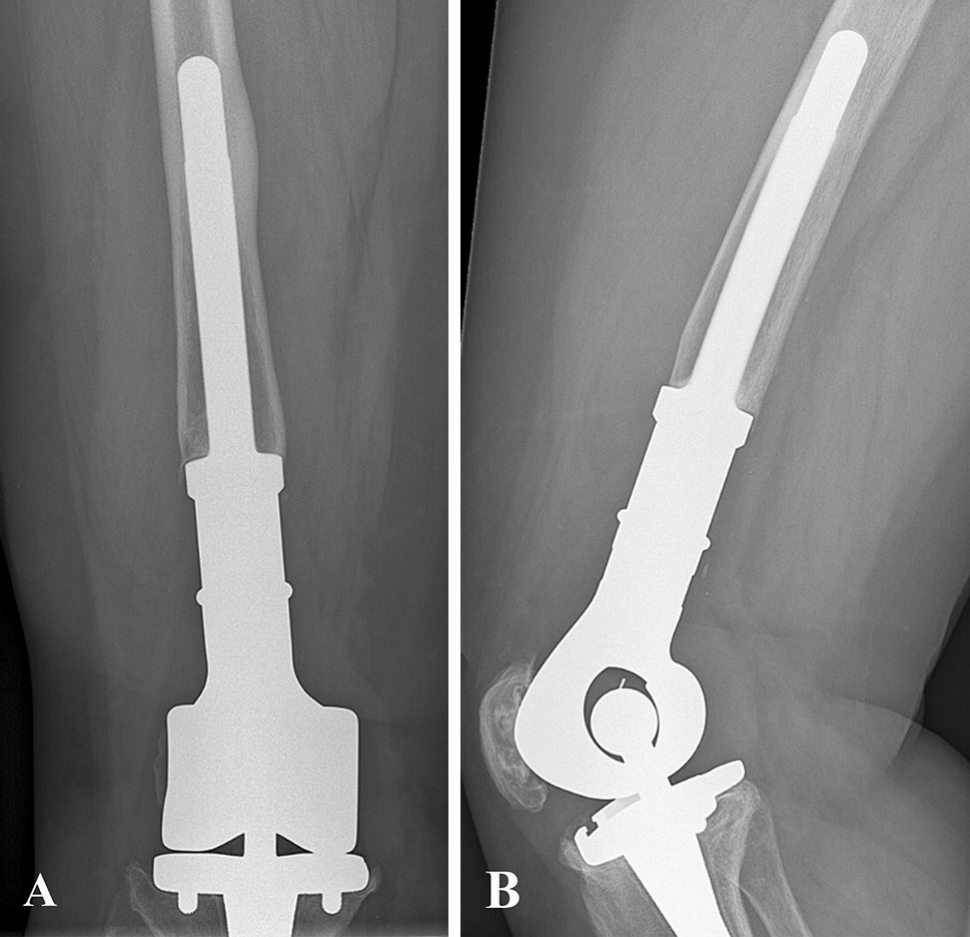
Conventional AP (**A**) and lateral (**B**) radiographs taken 6 years after extraarticular resection for an osteosarcoma of the distal femur in a 46-year-old female patient. The defect was reconstructed with an uncemented HA-coated MUTARS^®^ distal femoral replacement with a PEEK-OPTIMA^®^ locking mechanism. The postoperative course was uncomplicated and no further procedures were undertaken.

Eighty-nine reconstructions (81%) were distal femoral replacements and 21 (19%) were proximal tibial replacements. Eleven distal femoral replacements (11 of 89 [12%]) had a cemented femoral stem. Of 78 uncemented distal femoral replacements (78 of 89 [88%]), 42 were HA-coated (42 of 78 [54%]). All proximal tibial replacements had an uncemented tibial stem, 12 of which were HA-coated (12 of 19 [57%]) (Fig. 1A–B); one (one of 21 [5%]) had a cemented femoral stem. Patellar components were used in 37 distal femoral replacements (37 of 89 [42%]) and in three proximal tibial replacements (three of 21 [14%]). Median total resection length was 16 cm (range, 12–30 cm) for distal femoral replacements and 14 cm (range, 12–26 cm) for proximal tibial replacements. Attachment tubes were used in 14 proximal tibial replacements (14 of 21 [67%]) and in two distal femoral replacements (two of 89 [2%]). An extensor reconstruction was performed in 11 proximal tibial replacements (11 of 21 [58%]) and six distal femoral replacements (six of 89 [7%]). Rotation of a gastrocnemius muscle flap was performed in four proximal tibial replacements (four of 21 [19%], in one case combined with a split skin graft). Allogeneic fascia lata were used in six distal femoral replacements (six of 89 [7%]) and in two proximal tibial replacements (two of 21 [10%]). Three implants (three of 110 [3%]) were silver-coated.

During tumor resection, clear surgical margins were obtained in 95 patients (95 of 101 [94%]). Two patients (two of 101 [2%]) with giant cell tumors had intentional intralesional surgery. Four patients (four of 101 [5%]) had contaminated margins.

## Results

### Mechanical Complications

Complications of soft tissue or instability (Henderson Type 1) occurred in seven reconstructions (seven of 110 [6%], six distal femoral replacements, one proximal tibial replacement) after a median of 5 months (range, 0–46 months). These complications included skin necrosis (n = 2 [two of 110 {2%}]), flexion contracture (n = 2 [two of 110 {2%}]), and patellar dislocation (n = 1 [one of 110 {1%}]). One patient underwent surgery for extensor mechanism insufficiency (n = 1 [one of 110 {1%}]). We could not identify factors associated with the occurrence of Type 1 complications. No Type 1 complication resulted in removal or revision of the prosthesis.

**Fig. 2:**
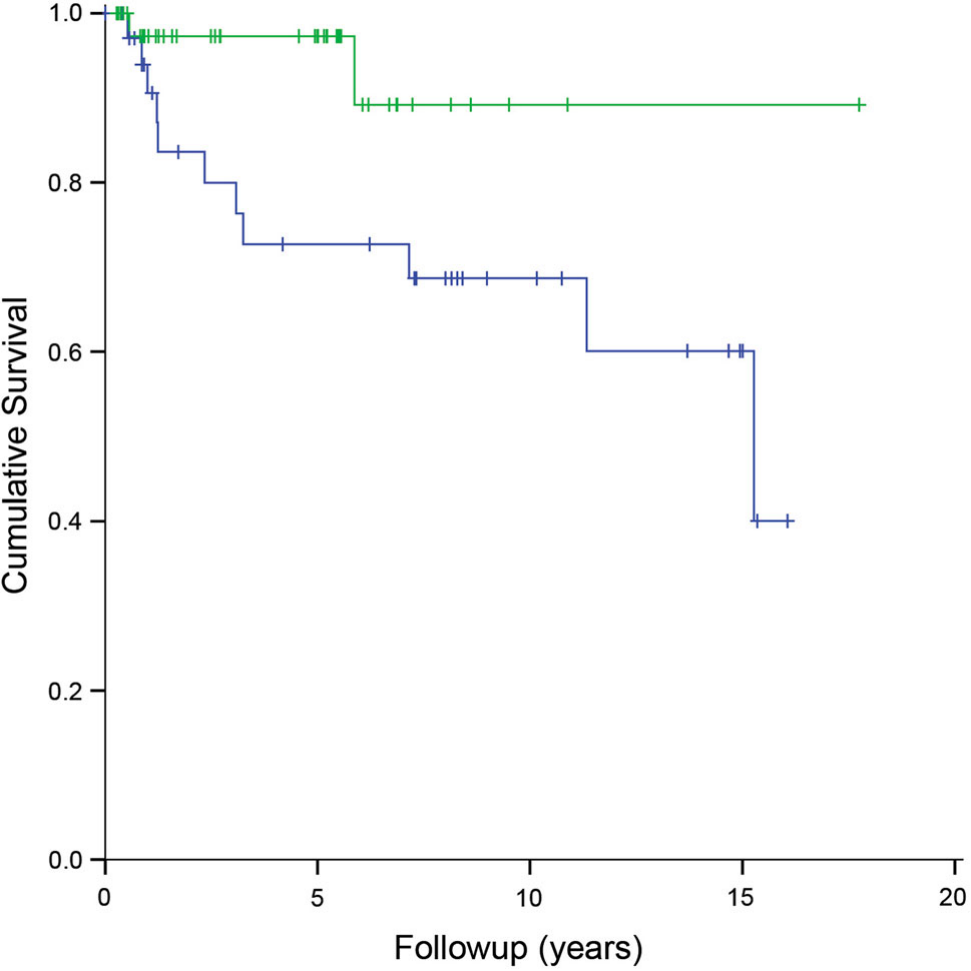
Kaplan-Meier curve showing survival to the occurrence of loosening for uncemented uncoated (blue line, n = 36) and uncemented HA-coated (green line, n = 42) distal femoral replacements.

Aseptic loosening (Henderson Type 2) occurred in 15 distal femoral replacements (15 of 89 [17%]) and two proximal tibial replacements (two of 21 [10%]) after a median of 1.2 years (range, 0.5–15 years). Both proximal tibial replacements had loosening of the femoral component (both uncemented, one HA-coated), for which cemented refixation was undertaken. Of the 15 distal femoral replacements, nine had loosening of the femoral component, three of the tibial component, and three of both components. Treatment consisted of cemented refixation (n = 6), uncemented revision of the femoral component (n = 4), cemented revision (n = 4), and a total femoral replacement (as a result of poor remnant host bone) (n = 1). With the numbers we had, for uncemented distal femoral replacements, we could not detect an association between reconstruction length and the rate of loosening (hazard ratio [HR], 1.06; 95% CI, 0.93–1.21; p = 0.393) nor a difference in loosening between revision (five of 17 [29%]) and primary reconstructions (eight of 61 [13%]) (HR, 1.72; 95% CI, 0.55–5.38; p = 0.354). Uncemented HA-coated distal femoral replacements had a lower risk of loosening (two of 42 [5%]) than uncemented uncoated implants (11 of 36 [31%]) (HR, 0.23; 95% CI, 0.05–1.06; p = 0.060) (Fig. 2).

Structural complications (Henderson Type 3) occurred in 15 reconstructions (15 of 110 [14%]) after a mean of 3 years (range, 0.0–13.5 years). These included six complications of the locking mechanism: three fractures, two instances of wear, and one unlocking of the locking mechanism. Four occurred in PEEK-OPTIMA^®^ locks. There were four periprosthetic fractures occurring at 3 weeks, 8 months, 20 months, and 6 years, respectively. There were three fractures of the femoral component, two with a 12-mm core diameter and a defect of 17.5 and 21.5 cm and one with a 16-mm core diameter stem with a defect of 15.5 cm. These stem fractures occurred 2, 4, and 4 years, respectively. There was one fractured insert and one implant rotation deformity.

Two prosthetic fractures and one periprosthetic fracture resulted in revision or removal of the entire implant; others were managed either conservatively or with limited revision procedures such as fixation of the periprosthetic fracture with a small plate, relocking of the locking mechanism, or revision of the locking mechanism. In addition, undisplaced fissure fractures occurred during implantation in 11 reconstructions: nine distal femoral replacements and two proximal tibial replacements. All healed uneventfully. Replacement of the bushings was performed in nine reconstructions (nine of 110 [8%]) after a mean of 6 years (range, 0.1–18 years).

### Nonmechanical Complications

Deep infections (Henderson Type 4) occurred in 15 reconstructions (15 of 110 [14%]). According to the Henderson classification, nine infections were early (< 2 years after implantation [nine of 110 {8%}]) and six were late (six of 110 [5%]). Three early-infected implants were retained. Three late infections occurred after operative intervention for another complication; of these, two were retained.

Local recurrences (Henderson Type 5) occurred in 10 patients (10 of 101 [10%]) after a mean of 2 years (range, 0.8–6 years). All patients who developed a local recurrence had clear surgical margins during the index resection. Two patients had received radiotherapy (one leiomyosarcoma, one high-grade osteosarcoma of an unusual subtype). Treatment consisted of ablative surgery in seven patients and of a second limb-salvaging resection (without removing the implant) in two. In one patient no further treatment was undertaken as a result of a poor prognosis. Focusing on patients without prior resections, local recurrences occurred in five of 39 patients with an extraarticular resection (13%) and in four of 45 patients with an intraarticular resection (9%) (p = 0.561).

### Implant Failure Rates

**Fig. 3:**
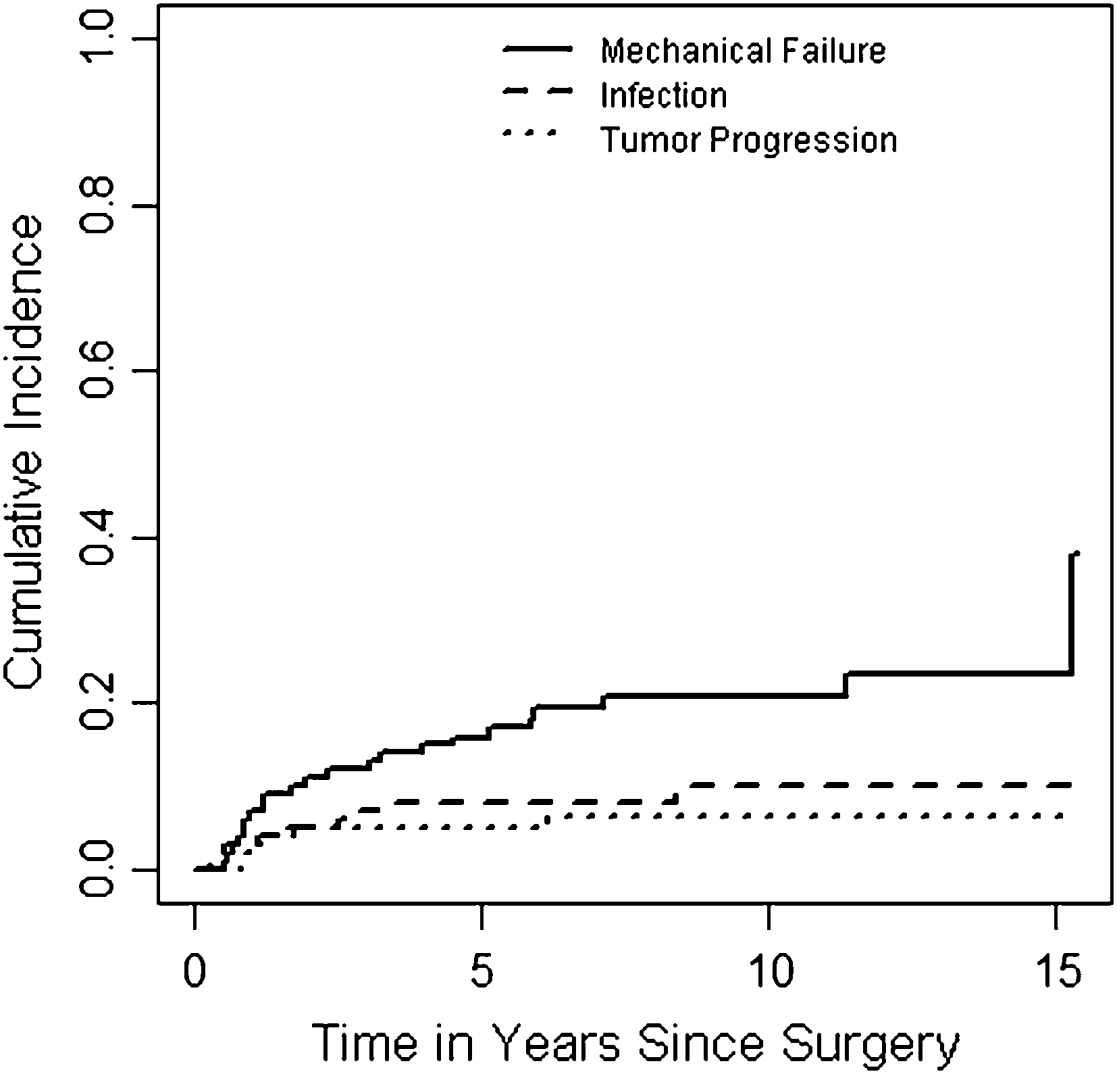
Competing-risk analyses of implant failure. This plot shows the cumulative incidence of mechanical failure (Type 1–3), infection (Type 4), and tumor progression (Type 5). Patient mortality was used as a competing event in these analyses.

With failure for mechanical reasons (Types 1–3) as the endpoint, the cumulative incidences of implant failure at 5, 10, and 15 years were 16.9% (95% CI, 9.6–24.2), 20.7% (95% CI, 12.5–28.8), and 37.9% (95% CI, 16.1–59.7), respectively (Fig. 3). With failure for infection (Type 4) as the endpoint, these were 7.9% (95% CI, 2.7–13.2), 10.0% (95% CI, 3.5–16.4), and 10.0% (95% CI, 3.5–16.4), respectively. With failure from tumor progression (Type 5) as the endpoint, these were 5.0% (95% CI, 0.7–9.2), 6.2% (95% CI, 1.4–11.0), and 6.2% (95% CI, 1.4–11.0), respectively . None of the assessed variables (extraarticular resection, HA coating of uncemented implants, reconstruction length of > 16 cm, adjuvant therapy, or having a preceding reconstruction) was found to have been associated with differences in implant survival in univariable Cox regression analyses.

### Limb Salvage

Limb salvage was achieved in 91 patients (90%). In total, 64 of 101 patients had their original MUTARS^®^ in situ without refixation, partial revision, or major revision/removal of the implant. Not all failures required a second MUTARS^®^ because some cases of failure were managed while the same implant was in place (for example the cases of loosening that were managed with cemented refixation of the implant or failure of the locking mechanism, which was managed with revision of the polyethylene lock). In all, 55 patients (55 of 101 [55%]) required a total of 141 further surgical procedures: 78 (78 of 141 [55%]) for infection and 42 (42 of 141 [30%]) for mechanical reasons. At review, 90 patients (90 of 101 [89%]) had a MUTARS^®^ in situ. Above-knee amputations were undertaken in seven patients (seven of 101 [7%]; five as a result of a local recurrence, two resulting from infection), rotationplasty in two (two of 101 [2%]; one as a result of local recurrence, one resulting from infection), total femoral replacement in one (one of 101 [1%], as a result of loosening and poor remnant host bone), and knee disarticulation in one (as a result of a periprosthetic fracture).

## Discussion

Modular endoprostheses are frequently used to reconstruct skeletal and knee defects created by resecting a bone neoplasm from the distal femur or proximal tibia. However, they are associated with substantial complication rates on both the short and long term, most notably infection and aseptic loosening [19, 26, 27]. We sought to evaluate the long-term results of knee arthroplasty with MUTARS^®^ modular endoprostheses in the treatment of primary tumors, emphasizing on mechanical complications.

Our study has a number of limitations. Preferably, one would report on proximal tibial and distal femoral replacements separately because they may differ in the types of complications by site. However, we were hampered by a limited number of patients and we therefore chose to report on knee arthroplasty as one group. We grouped patients who had a previous reconstruction together with those reconstructions done for a primary resection and these groups are disparate, which might have influenced our overall risk of loosening. However, we feel that the results as now presented best describe our clinical experiences with this implant system during the period under study. Moreover, as a result of the long retrospective period of our study, we were unable to obtain functional outcome scores and quality-of-life scores. We had no comparison groups so we are unable to determine if this endoprosthesis offers advantages or disadvantages compared with other prostheses or types of reconstruction.

All complications of soft tissue and instability (Henderson Type 1) were managed without implant removal. Few studies specified the incidence of complications of soft tissue and instability; however, our results (6%) are comparable with those recently reported by others (7%–9%) [1, 28]. Pala et al. [28] noted that Type 1 complications were more frequent in primary than in revision reconstructions (10% versus 4%). Although with the numbers we had we could not demonstrate an association between having a previous reconstruction or an extraarticular resection, it is plausible that soft tissue problems occur more often in previously operated sites and after more extensive resections as a result of scarring and restricted flexibility of surrounding soft tissues. The most common Type 1 complication in a large study on KMFTR and HMRS knee replacements (Stryker, Newbury, UK) was patellar tendon rupture with an overall incidence of 5% [32]. We did not observe any patellar tendon ruptures. We attribute this to the use of the attachment tube. The tube allows for ingrowth of the extensor apparatus and apparently ensures reliable, long-lasting fixation [14].

Aseptic loosening (Henderson Type 2) occurred in 12% of the primary reconstructions. This is comparable with most long-term followup studies (Table 3). The high risk of loosening of megaprostheses around the knee has been ascribed to many factors, including the torque acting on the stems and the long lever arm associated with greater resection length [1, 35]. We could not demonstrate an influence of resection length in the current series. HA coating appeared to decrease the risk of loosening of uncemented distal femoral replacements. Pala et al. reported a comparable rate (6%) for uncemented HA-coated GMRS prostheses (Stryker, Rutherford, NJ, USA), although their followup was substantially shorter (Table 3). Satisfactory rates of loosening (0%–8%) have also been reported for cemented custom-made implants with HA collars (Stanmore Implants Worldwide, Elstree, UK) [8, 26, 27]. Although loosening may occur as late as 25 years after cemented fixation [19, 26, 27], it is unlikely to occur after bony ingrowth of a HA-coated implant has taken place [4]. A prerequisite for ingrowth is primary stability; relative motion of more than 150 μm between bone and stem is critical for adequate fixation [21]. Blunn et al. [4] reported on a series of uncemented tumor implants (Stanmore Implants Worldwide) and noted that subperiosteal cortical bone loss occurred at the midstem level. This process, however, stabilized, and none of their implants was revised as a result. We did not observe this as a reason for revision.

**Table 3:** Overview of literature on knee replacement in bone tumor surgery

Study	Number	Year of surgery	Implant type*	Followup (years)^†^	Site^‡^	Diagnoses^§^	Hinge Type^||^	Fixation method^¶^	Extraarticular resection	Aseptic loosening	Implant survival/cumulative incidence of failure
Pala et al. [28]	247	2003 – 2010	GMRS (Stryker)	4 (2–8)	DF 76% PT 25%	Prim. 98% Mets. 2%	RH	Unc-HA coated 90% Cem 9%	–	6%	70 and 58% at 4 and 8 years (survival, all failure modes)
Myers et al. [26]	335	1973 – 2000	Custom (Stanmore)	Survivors: 12 (5–30) Deceased: N/R	DF	Prim. 94% Mets. 6%	FH 48% RH 52%	Unc-HA collar 4% Cem-HA collar 43% Cem 53%	Rarely	FH: 35% at 10 years RH: -	83%, 67%, and 51% at 5, 10, and 15 years, respectively (survival, as a result of aseptic loosening, fracture of the implant, infection, breakage, etc)
Myers et al. [27]	194	1977 – 2002	Custom (Stanmore)	Survivors: 14.7 (5–29) Deceased: N/R	PT	Prim. 94% Mets. 6%	FH 49% RH 51%	Cem/Cem-HA collar (N/R)	Rarely	FH: 46% at 10 years RH: 3%	79%, 58%, and 45% at 5, 10, and 15 years, respectively (survival, as a result of aseptic loosening, breakage, infection, etc)
Kinkel et al. [22]	77	1995 – 2005	MUTARS (implantcast)	3.8 (0.3–10.7)	DF 64% PT 36%	Prim. 90% Mets. 10%	RH	Unc 78% Cem 22%	40%	17%	57% at 5 years (survival, reasons N/R)
Griffin et al. [15]	99	1989 – 2000	KMFTR (Stryker)	Med. 6.1 (0.3–13.2)	DF 75% PT 36%	Prim.	FH	Unc	13%	2%	N/R for overall population
Biau et al. [2]	91	1972 – 1994	Custom (Stryker)	Med. 5.2 (0.0–28.6)	DF 62% PT 38%	Prim. 98% Mets. 2%	FH	Cem	3%	20%	76%, 45%, and 29% at 5, 10, and 15 years, respectively (survival, revision for any reason)
Bickels et al. [3]	110	1990 – 1998	Modular 66% Custom 25%* (Howmedica)	Med. 7.8 (2–16.5)	DF	Prim. 98% Non-tum. 2%	FH 7% RH 93%	Cem	2%	5%	93% and 88% at 5 and 10 years, respectively, overall survival
Morgan et al. [24]	105	1985 – 2004	Modular (different manufacturers)	Med. 4.8 (0.1–19.6)	DF 72% PT 28%	N/R	RH	Cem	–	17%	73% and 59% at 5 and 10 years, respectively (survival, failure modes 1–4)
Plotz et al. [29]	60	1976 – 1996	Custom (different manufacturers)	4.9 (0.1–19.1)	DF 75% PT 25%	Prim. 83% Mets. 17%	N/R	Hybrid 5% Unc-Pc 45% Cem 50%	N/R	5%	34% and 25% at 5 and 10 years, respectively (survival of the prostheses without revision surgery)
Ruggieri et al. [32]	669	1983 – 2006	KMFTR/HMRS (Stryker)	11 (2–25)	DF 71% PT 24% TF 3% EAK 1%	Prim. 97% Mets. 3%	FH	Unc 91% Cem 9%	1%	6%	80% and 55% at 10 and 20 years, respectively (survival, breakage, aseptic loosening, or infection)
Coathup et al. [8]	61	1992 – 2001	Custom (Stanmore)	8.5 (2–18)	DF	Primary	RH	Cem-HA collar	N/R	8%	75%, 84%, and 89% at 5, 10, and 15 years, respectively (survival, all failure modes)
Batta et al. [1]	69	1994 – 2006	Custom (Stanmore)	10.4 (0.3–17.7)	DF	Primary	RH	Unc-HA collar	N/R	13%	73%, 65%, and 55% at 5, 10, and 15 years, respectively (survival, all failure modes)
Schwartz et al. [33]	186	1980 – 2008	Custom 54% GMRS 46% (different manufacturers)	8.0 (0.1–28.0)	DF	Prim. 98% Mets. 2%	RH	Cem/Cem-Pc collar (N/R)	N/R	12%	77% at 10 years (survival, revision of stemmed components for all failure modes)
Current study	110	1995 – 2010	MUTARS (implantcast)	Overall: 7.2 (0.4–18.0) Survivors: 9.5 (5.0–18.0) Deceased: 3.1 (0.4–14.1)	DF: 81% PT: 19%	Primary	RH	Unc-uncoated 41% Unc-HA coated 49% Cem 10%	46%**	Primary reconstructions: 12% Overall: 15%	Cumulative incidence of failure for mechanical reasons (Types 1–3): 17%, 21%, and 38% at 5, 10, and 15 years Cumulative incidence of failure for infection (Type 4): 8%, 10%, and 10% at 5, 10, and 15 years, respectively Cumulative incidence of failure for tumor progression (Type 5): 5%, 6%, and 6% at 5, 10, and 15 years, respectively

* Implant type: GMRS = Global Modular Replacement System (Stryker, Rutherford, NJ, USA); Custom = custom-made, different manufacturers; MUTARS = Modular Universal Tumor and Revision System (implantcast, Buxtehude, Germany); KMFTR = Kotz Modular Femur Tibia Reconstruction (Stryker, Rutherford, NJ, USA); Mod. = modular, different manufacturers; HMRS = Howmedica Modular Reconstruction System (Stryker, Rutherford, NJ, USA).

^†^mean followup, unless otherwise stated (med. = median) with the range in parentheses; ^‡^DF = distal femur, PT = proximal tibia, TF = total femur, EAK = extraarticular knee; ^§^prim. = primary tumor, mets. = metastatic disease, non-tum. = nontumorous; ^||^RH = rotating hinge, FH = fixed hinge; ^¶^unc = uncemented, cem = cemented, Pc = porous-coated; **of the patients in whom the MUTARS^®^ was implanted during primary surgery; N/R = not reported.

Like most modern tumor prostheses, the implants used in our study had a rotating hinge (Table 3). Authors postulated that rotating hinges reduce the risk of bushing wear and of loosening, the latter by reducing torsional stresses at the implant-bone interface [13, 27, 28]. Myers et al. [27] reported a reduction in loosening rates after the introduction of rotating hinges, although it is unclear whether this reduction should be ascribed to the rotating hinge, the HA-coated collar, or a combination of both [26]. We are of the opinion that uncemented HA-coated implants with a rotating hinge offer the best possibility to achieve stable fixation and therefore durable results, although we cannot definitively support this contention from our results. Loosening appeared to be a particular problem in those implants that were used as a revision of a previously failed reconstruction. Foo et al. [11] discussed the difficulties encountered with the use of uncemented MUTARS^®^ prostheses after failed allograft reconstructions. We concur with their conclusion that cemented fixation is preferred in case of poor remnant bone quality as may be the case after allograft reconstruction or loosened endoprostheses.

Structural complications (Henderson Type 3) occurred in 15%. Introduction of the PEEK-OPTIMA^®^ lock has not resulted in a reduction of long-term structural complication rates. Since 2010, we routinely use the MUTARS^®^ metal-on-metal locking mechanism because we believe this mechanism should be able to better withstand the high mechanical stresses. Our prosthetic fracture rate (3%) is comparable with the rate reported by Myers et al. (2%) [26] and compares favorably with other studies (5%–7%) [2, 15, 24], whereas our followup is among the longest reported in the literature (Table 3). All three fractured implants had a total resection length of ≥ 15.5 cm and two had 12-mm stems. Previously, Gosheger et al. [13] reported stem fractures in four MUTARS^®^ reconstructions, all with a stem diameter of 12 mm or less. We believe that careful reaming and implantation of the largest possible stem diameter are advisable to reduce the risk of stem fractures and recommend using stems of at least 12 mm.

Infection (Henderson Type 4) occurred in 13% and resulted in removal of the implant in 9%, which is comparable with most previous studies (6%–20%) [2, 15, 26–28, 32]. We could not demonstrate a difference among early and late infections with regard to the possibility of implant retention. However, three late infections occurred after operative intervention for another complication; such infections may be treated as an acute infection as opposed to late-occurring low-grade infections. Currently, we routinely use silver-coated implants, which may reduce the risk of infection and increase the likelihood of being able to retain the implant in case it gets infected [13, 36]. Others previously reported a reduction in the frequency of infection since the routine use of muscle flaps [27].

Failure as a result of local recurrence (Type 5 complication) occurred in 7%. Other long-term followup studies reported comparable rates (5%–6%) [3, 15, 26, 27]. Kinkel et al. [22] noted that the rate of extraarticular resection was substantially higher in their population (40%) compared with other series (0%–13%; Table 3). With the numbers we had, we found no difference in relapse or complication risks between intra- and extraarticular resections. On the other hand, others reported that extraarticular resection is associated with an increased risk of infection and loosening [13, 16]. One may therefore question whether the high rate of extraarticular resection (46% of the primary reconstructions in our study) is truly justified. Careful evaluation of joint involvement with use of modern imaging techniques (PET-CT, gadolinium-enhanced MRI) may aid to avoid unnecessary extraarticular resections.

As a result of the fact that nearly all studies have used Kaplan-Meier survival analyses to compute implant survival rates, and because different classifications and definitions of failures have been used, it is difficult to adequately compare implant failure rates. Nevertheless, our long-term cumulative incidence rates of failure appear to be comparable to those reported by others [1, 24, 28] and compare favorably with others [2, 22, 26, 27] (Table 3).

Despite needing more operative procedures for complications, we were able to achieve limb salvage in 90% of our patients. The majority of our patients had a MUTARS^®^ (but not necessarily the original MUTARS^®^ implant) in situ at latest followup, indicating that most complications could be adequately managed.

Although no system has yet proved ideal to restore normal function and demonstrate long-term retention of the implant, MUTARS^®^ modular endoprostheses represent a reliable long-term option for knee replacement after tumor resection, which seems to be comparable to other modular implants available to surgeons. The cumulative incidence of implant failure was 20.7% at 10 years with mechanical failure as the endpoint. Aseptic loosening was the most important mechanical complication. HA coating of uncemented implants may reduce the risk of loosening, and we currently use uncemented HA-coated implants believing that it is optimal for durable fixation. We conclude that MUTARS^®^ represents a reliable system with long-term results comparable to other prostheses and types of reconstructions for tumor resections about the knee.
